# DNA methylome analysis identifies BMI‐related epigenetic changes associated with non‐small cell lung cancer susceptibility

**DOI:** 10.1002/cam4.3906

**Published:** 2021-05-03

**Authors:** Guyanan Li, Hua Meng, Yansen Bai, Wei Wei, Yue Feng, Mengying Li, Hang Li, Meian He, Xiaomin Zhang, Sheng Wei, Yangkai Li, Huan Guo

**Affiliations:** ^1^ Department of Occupational and Environmental Health Key Laboratory of Environment & Health, Ministry of Education; State Key Laboratory of Environmental Health (Incubating) School of Public Health Tongji Medical College Huazhong University of Science and Technology Wuhan Hubei China; ^2^ Department of Epidemiology and Biostatistics School of Public Health Tongji Medical College Huazhong University of Science and Technology Wuhan Hubei China; ^3^ Department of Thoracic Surgery Tongji Hospital Tongji Medical College Huazhong University of Science and Technology Wuhan Hubei China

**Keywords:** BMI, DNA methylation, genome‐wide methylation association study, mediation analysis, non‐small cell lung cancer

## Abstract

**Background:**

Body mass index (BMI) has been reported to be inversely associated with incident risk of non‐small cell lung cancer (NSCLC). However, the underlying mechanism is still unclear. This study aimed to investigate the role of DNA methylation in the relationship between BMI and NSCLC.

**Methods:**

We carried out a genome‐wide DNA methylation study of BMI in peripheral blood among 2266 Chinese participants by using Illumina Methylation arrays. For the BMI‐related DNA methylation changes, their associations with NSCLC risk were further analyzed and their mediation effects on BMI‐NSCLC association were also evaluated.

**Results:**

The methylation levels of four CpGs (cg12593793, cg17061862, cg11024682, and cg06500161, annotated to *LMNA*, *ZNF143*, *SREBF1*, and *ABCG1*, respectively) were found to be significantly associated with BMI. Methylation levels of cg12593793, cg11024682, and cg06500161 were observed to be inversely associated with NSCLC risk [OR (95%CI) =0.22 (0.16, 0.31), 0.39 (0.30, 0.50), and 0.66 (0.53, 0.82), respectively]. Additionally, cg11024682 in *SREBF1* and cg06500161 in *ABCG1* mediated 45.3% and 19.5% of the association between BMI and decreased NSCLC risk, respectively.

**Conclusions:**

In this study, we identified four DNA methylation sites associated with BMI in the Chinese populations at the genome‐wide significant level. We also found that the BMI‐related methylations of *SREBF1* and *ABCG1* could mediate about a quintile‐to‐half of the effect of BMI on reduced NSCLC risk, which adds a potential mechanism underlying this association.

## Graphical abstract

We identified four BMI‐related CpGs (located at *LMNA*, *ZNF143*, *SREBF1*, and *ABCG1*) in Chinese populations. The methylation levels of three CpGs (located at *LMNA*, *SREBF1*, and *ABCG1*) in whole blood were inversely associated with lung cancer risk. The cg11024682 and cg06500161 annotated to *SREBF1* and *ABCG1* genes, could mediate the associations of BMI and lung cancer risk.

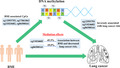

## 1 INTRODUCTION

The prevalence of overweight and obesity has increased substantially in the past three decades.^1^ In China, the overall prevalence of adult overweight and obesity was 30.1% and 11.9%, respectively, in 2012.^2^ Obesity is usually considered an oncogenic factor and over 20% of cancers are obesity‐related, including cancers of the liver, colon, and ovary.^3^ However, an inverse association between body mass index (BMI) and incident risk of non‐small cell lung cancer (NSCLC) has been established among both Caucasian and Asian populations.^2^, ^4^ Although this observation may reflect reverse causality related to the latent effect of lung cancer on BMI and confounding effect of tobacco smoking,^5^ some prospective cohort studies confirmed the inverse association among never smokers or after excluding cases diagnosed at early follow‐up years.^6^, ^7^ A previous meta‐analysis, including 20 cohort studies and 11 case‐control studies, found that among never smokers, excess body weight (BMI ≥25 kg/m^2^) was inversely associated with incident lung cancer risk compared with normal weight (BMI: 18.5–24.9 kg/m^2^) [risk ratio = 0.83; 95% confidence interval (95% CI) = 0.70–0.98].^8^ Nevertheless, there were no clear explanations about the underlying mechanisms.

DNA methylation, the well‐known epigenetic mechanism, is sensitive to environmental exposure.^9^ DNA methylation alterations in peripheral blood related to BMI have gained much attention from research groups.^10^, ^11^ Mendelson *et al* conducted an epigenome‐wide association study (EWAS) of BMI and found differential methylations at 83 CpGs in peripheral blood among 7798 European populations.^12^ Another EWAS of BMI, contained 6925 European and 3336 Indian‐Asian individuals, identified 187 BMI‐related CpG markers.^13^ DNA methylation is also involved in carcinogenesis and progression of lung cancer, mainly via regulating the expression of genes and impeding the stability of genome.^14^, ^15^ It was reported that the whole blood methylation levels of smoking‐related genes, such as *AHRR* and *F2RL3*, were associated with lung cancer risk [OR (95% CI) = 0.37 (0.31, 0.54) and 0.40 (0.31, 0.56), respectively].^16^

With the development of environmental epigenetics, many researchers focus on the obesity‐epigenetics‐cancer risk axis.^17^ Obesity‐associated reprogramming of the epigenome via DNA methylation may alter the expression of genes that promote or inhibit tumor progression.^18^ Frederick et al showed that obesity played a protective role against premenopausal breast tumorigenesis by increasing *Line*‐*1* DNA methylation level and expression of tumor suppressor gene *SFRP1* in breast tissues.^19^ Nagashima et al reported that the dysregulation of DNA methylation in endometrial epithelial cells resulted in endometrial cancer development in women with obesity.^20^ Similarly, the BMI‐related methylated genes (*ZNF543* and *ZNF397OS*) were found to be differentially epigenetically regulated in colorectal cancer tissues.^21^ The methylations of *ABCG1* and *SREBF1* in whole blood were well‐known to be associated with obesity.^12^ More importantly, these two genes, as potential oncogenes of lung cancer, have been found to be implicated in the proliferation and apoptosis of lung cancer cells.^22^, ^23^ However, the role of DNA methylation in the relationship between BMI and lung cancer remains largely unknown.

In this study, we first examined the blood methylation profiles with BMI by using a 2‐stage EWAS design among the Chinese populations. For the BMI‐related CpG sites, we further assessed their associations with NSCLC risk and explored their mediation effects on the BMI‐lung cancer association in two NSCLC case‐control studies.

## 2 METHODS

### 2.1 Study populations

**FIGURE 1:**
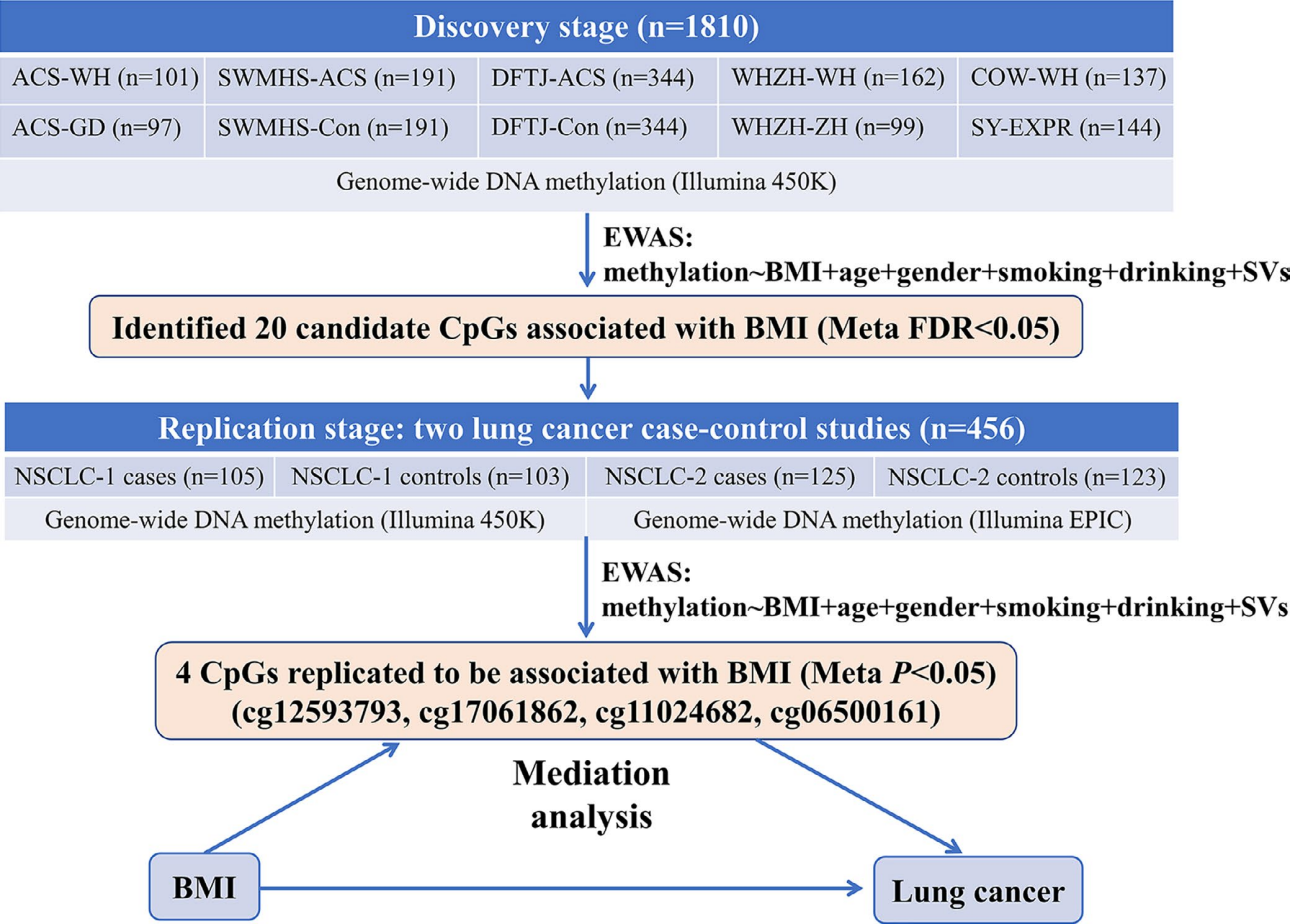
Flow chart of study design

We performed a 2‐stage EWAS in a total of 2266 participants from 14 sub‐studies to identify BMI‐related CpGs (Figure 1).

The discovery stage comprised 1810 individuals from 10 studies: the acute coronary syndrome (ACS) patients from Wuhan and Guangdong Province (abbreviated as WH‐ACS and GD‐ACS, respectively), Wuhan and Zhuhai residents of Wuhan‐Zhuhai Cohort Study (abbreviated as WHZH‐WH and WHZH‐ZH, respectively), workers from the Coke‐Oven Cohort Study (abbreviated as COW‐WH), nested ACS case‐controls from the Dongfeng‐Tongji Cohort Study (abbreviated as DFTJ‐ACS and DFTJ‐Con, respectively), nested ACS case‐controls from the Shanghai Women's and Men's Healthy Study (abbreviated as SWMHS‐ACS and SWMHS‐Con, respectively), and subjects for investigating methylation‐expression correlations from Shiyan, China (abbreviated as SY‐EXPR). Detailed information about these subjects has been described previously.^24^, ^25^

The validation stage consisted of two NSCLC case‐control studies (abbreviated as NSCLC‐1 and NSCLC‐2), including 109 pairs and 126 pairs of NSCLC cases and controls, respectively, who were matched at age (±2 years‐old) and gender. NSCLC patients in NSCLC‐1 and NSCLC‐2 underwent surgical resection of primary lung cancer from June 2012 to December 2014 and June 2018 to December 2019, respectively, in the Department of Thoracic Surgery, Tongji Hospital in Wuhan, China. The diagnosis of NSCLC was based on pathological examination from surgical pathology archives of the Tongji Hospital. Patients who had been treated with preoperative chemoradiotherapy and biotherapy or suffered from other lung diseases simultaneously, such as tuberculosis, pneumonia, and silicosis were excluded. The peripheral blood samples of NSCLC patients were collected before the surgical operation. The matched controls, who were free of malignant cancer, diabetes, stroke, and cardiovascular diseases, were selected from the physical examination center, Tongji Hospital, during the same periods. After quality control (QC), 105 cases, including 54 of lung adenocarcinoma (LUAD) and 51 of lung squamous carcinoma (LUSC) and 103 controls were retained in NSCLC‐1, and 125 cases (including 78 of LUAD and 47 of LUSC) and 123 controls were kept in NSCLC‐2.

The 144 subjects in the SY‐EXPR study, as mentioned above, who had a regular physical examination at the Department of Health Examination in Sinopharm Dongfeng General Hospital in Shiyan, China during April and May of 2015, were recruited to investigate the transcriptional regulating effects of DNA methylation on gene expression.^24^ These participants, ranged from 20 to 70 years old, were free of infectious conditions within 2 weeks of the examination and did not take any medicine in the past month before the physical examination.

The detailed descriptions are provided in Supplementary Materials. All participants in this study gave their written informed consent.

### 2.2 The measurement of BMI and covariates

At recruitment, weight and height were measured by trained examiners with participants standing without shoes. BMI was calculated as weight divided by height squared (kg/m^2^). Demographic characteristics were collected from face‐to‐face interviews. Subjects who had smoked >1 cigarette per day for >1 year were defined as current smokers; those who ever smoked and had quitted over half a year were defined as former smokers; otherwise, they were defined as never smokers. Subjects who had drunk alcohol at least once a week for more than half a year were defined as current alcohol drinkers; those who had ever drunk alcohol but quitted over half a year were defined as former alcohol drinkers; otherwise, they were defined as never alcohol drinkers.^26^ We combined current and former smokers into ever smokers, and combined current and former alcohol drinkers into ever‐drinkers. The peripheral counts of white blood cells and the subtypes (including neutrophils, lymphocytes, monocytes, eosinophils, and basophils) were assessed by automated particle counters in the local laboratory.

### 2.3 Laboratory assays

DNA and RNA were extracted from peripheral blood using the BioTeke Whole Blood DNA Extraction Kit. Bisulfite conversion was performed using Zymo EZ DNA Methylation Kit (Zymo Research, Orange, CA). Methylation of genomic DNA was quantified by the Illumina Human Methylation 450 K array (Illumina, Inc., Boston, USA) for participants in the discovery stage as well as NSCLC‐1 study in the validation stage or Illumina Human Methylation EPIC array for participants in the NSCLC‐2 study in the validation stage. After QC and normalization, 430,302 CpGs in the discovery stage, and 433,439 and 808,064 CpGs in NSCLC‐1 and NSCLC‐2, respectively, were eligible for further analyses. For the 144 participants in the SY‐EXPR study, gene expression profiles were assayed using HumanHT‐12 v4 BeadChip (Illumina). Laboratory procedures, data processing, QC, and normalization are provided in detail in Supplementary Materials.

### 2.4 Statistical analyses

The surrogate variable analysis (SVA) was used to identify and remove potential effects of batches, cell compositions, and other unwanted sources of variation.^27^ Variables considered in SVA included age, gender, smoking status (coded as 0 and 1 for never and ever smokers, respectively), drinking status (coded as 0 and 1 for never and ever alcohol drinkers, respectively), and BMI (kg/m^2^, as a continuous variable). To eliminate outliers and archive a normal distribution, β value of each CpG was inverse normal transformed (INT, to a normal distribution with a mean value of 0 and an SD of 1): *CpG_INT_* = qnorm(rank(*CpG*)/length(*CpG*) + 1), mean = 0, SD = 1). The multivariate linear regression model was conducted independently in each sub‐study, with natural logarithm (ln) transformed BMI as the independent variable and the INT β value of each CpG as the dependent variable, with adjustment for age, gender, smoking status, drinking status, and all surrogate variables. Results in discovery and replication stages were combined by using a fixed‐effect meta‐analysis. We used false discovery rate (FDR) <0.05 to define epigenome‐wide meta‐analysis significance in the discovery stage and *p* value for the association between BMI and DNA methylation <0.05 was used to define significance in the validation stage. Regional associations were performed by LocusZoom (http://locuszoom.sph.umich.edu/) for the associations of BMI with all nearby (±600 kbps) methylation sites of the replicated BMI‐related CpGs. The enrichment for the nominally significant association (*p *< 0.05) was evaluated by a 10,000‐permutation test. We annotated CpGs to the nearest genes using the annotation files provided by Illumina. Genes harboring or neighboring the replicated BMI‐related CpGs were considered in the further analyses. The correlations between CpGs methylation and gene expression levels (both values were INT) were assessed using linear regression models, with adjustment for age and gender.

Associations between the methylation levels of BMI‐related CpGs and NSCLC risk were further estimated in NSCLC case‐control studies by using multivariate logistic regression models, with adjustment for age, gender, smoking status, drinking status, and BMI. Blood counts major leukocytes were adjusted in the sensitivity analysis to confirm the above associations. The association heterogeneity between two NSCLC subtypes (LUAD and LUSC) was testified by regression with subtype dummy variables.

To evaluate the effects of DNA methylation on the association of BMI with NSCLC, a causal mediation analysis was conducted based on two models: (1) the mediator model, with the INT methylation level as the outcome and BMI as the predictor, with adjustment for age, gender, smoking status, and drinking status; (2) the outcome model, with lung cancer status as the outcome and BMI as the predictor, with adjustment for the methylation level of each CpG (mediator) and all covariates above. Natural indirect effect (NIE) and natural direct effect (NDE) were estimated on the OR scale by “%mediation” SAS macro, which was appropriate for a dichotomous outcome.^28^ Total effect (TE) was decomposed into NIE and NDE: TE = NIE × NDE (on the OR scale), and the proportion mediated by DNA methylation was calculated by the ratio between NIE and the sum of NIE and NDE (on the log OR scale).^28^ All analyses were performed using R version 3.6.1 or SAS version 9.4 (SAS Institute, Carry, NC), except for the meta‐analysis which was performed by METAL.^29^

## 3 RESULTS

### 3.1 Basic characteristics of the study populations

The present EWAS of BMI contained a total of 2266 Chinese individuals, including 1810 subjects from 10 sub‐studies in the discovery stage and 456 subjects from two NSCLC case‐control studies in the validation stage. Clinical characteristics of these participants are presented in Table 1. The mean ± SD of age among all participants ranged from 41.2 ± 10.3 years in the SY‐EXPR study to 64.6 ± 6.2 years in DFTJ‐ACS and DFTJ‐Con. The majority of subjects were males (64.2%). Mean ± SD of BMI ranged from 22.7 ± 2.3 kg/m^2^ in the WHZH‐ZH study to 25.3 ± 3.6 kg/m^2^ in the SWMHS‐ACS study.

**TABLE 1:** The general characteristics of study populations in the discovery and replication stages [*n* (%) or mean ±SD]

Variables	Discovery stage (*n* = 1810)										Replication stage (*n* = 456)			
	ACS‐WH	ACS‐GD	WHZH‐WH	WHZH‐ZH	COW‐WH	SWMHS‐ACS	SWMHS‐Con	DFTJ‐ACS	DFTJ‐Con	SY‐EXPR	NSCLC−1 cases	NSCLC−1 controls	NSCLC−2 cases	NSCLC−2 controls
*N*	101	97	162	99	137	191	191	344	344	144	105	103	125	123
Age	59.0 ± 10.2	59.4 ± 11.5	50.4 ± 12.9	59.5 ± 11.3	46.5 ± 8.9	61.5 ± 8.7	61.5 ± 8.6	64.6 ± 6.2	64.6 ± 6.2	41.2 ± 10.3	57.8 ± 8.2	57.9 ± 8.3	59.2 ± 8.0	59.5 ± 7.7
Male	81 (80.2%)	78 (80.4%)	126 (77.8%)	80 (80.8%)	107 (78.1%)	102 (53.4%)	102 (53.4%)	179 (52.0%)	179 (52.0%)	107 (74.3%)	75 (71.4%)	72 (69.9%)	84 (67.2%)	82 (66.7%)
Ever Smoking	63 (62.4%)	53 (54.6%)	88 (54.3%)	46 (46.5%)	87 (63.5%)	77 (40.3%)	65 (34.0%)	132 (38.4%)	115 (33.4%)	47 (32.6%)	65 (61.9%)	47 (45.6%)	71 (56.8%)	53 (43.1%)
Ever Alcohol Drinking	23 (22.8%)	20 (20.6%)	74 (45.7%)	12 (12.1%)	53 (38.7%)	37 (19.4%)	39 (20.4%)	91 (26.5%)	104 (30.2%)	55 (38.2%)	33 (31.4%)	40 (38.8%)	36 (28.8%)	59 (48.0%)
BMI	24.8 ± 2.8	23.0 ± 2.4	23.8 ± 2.9	22.7 ± 2.3	23.5 ± 2.7	25.3 ± 3.6	24.9 ± 3.8	25.0 ± 3.2	24.7 ± 3.0	24.2 ± 2.7	23.5 ± 3.2	25.1 ± 3.1	23.6 ± 2.9	23.9 ± 2.8
Counts of white blood cells, 10^9^/L														
Total white blood cells	7.3 ± 2.4	10.5 ± 3.8	5.9 ± 1.5	6.2 ± 1.8	6.7 ± 1.5	–	–	6.5 ± 1.6	5.8 ± 1.5	6.0 ± 1.4	6.7 ± 1.9	6.2 ± 2.0	6.5 ± 1.9	5.7 ± 1.8
Neutrophils	4.7 ± 2.3	7.8 ± 3.6	3.4 ± 1.0	3.3 ± 1.3	4.0 ± 1.2	–	–	3.8 ± 1.3	3.3 ± 1.1	3.5 ± 1.0	4.4 ± 1.8	3.7 ± 1.6	4.0 ± 1.6	3.4 ± 1.4
Lymphocytes	1.9 ± 0.7	1.8 ± 1.2	2.1 ± 0.6	2.4 ± 0.7	2.4 ± 0.6	–	–	2.1 ± 0.8	1.9 ± 0.6	2.0 ± 0.6	1.6 ± 0.5	1.8 ± 0.6	1.7 ± 0.6	1.7 ± 0.6
Intermediate cells	0.6 ± 0.3	0.8 ± 0.5	0.4 ± 0.3	0.5 ± 0.2	0.2 ± 0.1	–	–	0.7 ± 0.2	0.6 ± 0.2	0.6 ± 0.2	0.7 ± 0.3	0.7 ± 0.4	0.7 ± 0.3	0.7 ± 0.4

Note

Values were shown as mean ±SD or n (%). The intermediate cells were defined as the sum of monocytes, eosinophils, and basophils.

### 3.2 Epigenome‐wide association study of BMI

In the epigenome‐wide meta‐analysis in the discovery stage, methylation levels of 20 CpGs were associated with BMI after correction for multiple testing at a genome‐wide significance level (FDR < 0.05, genomic inflation factor λ = 1.06) (Figure S1). The regression coefficients of these CpGs and BMI in each sub‐study are provided in Table S1. Among them, the methylation levels of four CpGs (cg12593793, cg17061862, cg11024682, and cg06500161, annotated to *LMNA*, *ZNF143*, *SREBF1*, and *ABCG1*, respectively) were significantly associated with BMI in the validation stage (*p_meta_*_‐_ *_analysis_* < 0.05, Table S2). Meta‐analysis of both stages confirmed the robust negative associations of BMI with the methylation levels of cg12593793 and cg17061862 (β = −0.72, *p *= 1.23E‐13, and β = −0.92, *p *= 2.83E‐10, respectively), and the significant positive associations of BMI with the methylation levels of cg11024682 and cg06500161 (β = 1.07, *p *= 1.03E‐16, and β = 1.35, *p *= 1.61E‐17, respectively) (Table 2). To exclude the effect of smoking, we further examined the association of DNA methylation and BMI in never smokers. Although the links were weakened to some extent, the associations of DNA methylation levels at four CpGs (cg12593793, cg17061862, cg11024682, and cg06500161) with BMI were also statistically significant (Table S3). The regional methylation analysis indicated that methylation sites adjacent to the four BMI‐related CpGs showed enrichment of nominally significant associations with BMI (all *p*_enrichment_ < 0.05, Figure S2).

**TABLE 2:** The associations between BMI and 4 CpGs among subjects in the discovery stage, replication stage, and the meta‐analysis of both stages

Study populations	cg12593793 (*LMNA*)		cg17061862 (*ZNF143*)		cg11024682 (*SREBF1*)		cg06500161 (*ABCG1*)	
	β (SE)	*p*	β (SE)	*p*	β (SE)	*p*	β (SE)	*p*
Discovery stage (*n* = 1810)								
ACS‐WH	0.04 (0.45)	9.30E−01	−0.42 (0.65)	5.22E−01	1.10 (0.80)	1.71E−01	1.20 (0.96)	2.15E−01
ACS‐GD	−0.45 (0.50)	3.64E−01	−1.13 (0.73)	1.23E−01	−0.42 (0.92)	6.58E−01	−0.25 (0.99)	8.03E−01
WHZH‐WH	−0.24 (0.30)	4.32E−01	0.34 (0.43)	4.21E−01	1.73 (0.45)	2.05E−04	1.96 (0.65)	2.73E−03
WHZH‐ZH	−1.28 (0.48)	9.72E−03	−0.54 (0.75)	4.74E−01	1.16 (0.71)	1.09E−01	0.85 (1.00)	4.02E−01
COW‐WH	−0.81 (0.45)	7.14E−02	−1.77 (0.49)	4.44E−04	1.69 (0.58)	4.22E−03	0.82 (0.69)	2.41E−01
SWMHS‐ACS	−1.32 (0.37)	4.89E−04	−0.96 (0.56)	8.68E−02	1.15 (0.45)	1.11E−02	1.60 (0.57)	5.67E−03
SWMHS‐Con	−0.66 (0.30)	2.83E−02	−1.44 (0.48)	3.14E−03	0.94 (0.39)	1.66E−02	1.17 (0.49)	1.88E−02
DFTJ‐ACS	−1.18 (0.25)	4.24E−06	−1.15 (0.39)	3.76E−03	0.55 (0.31)	7.67E−02	1.92 (0.39)	1.47E−06
DFTJ‐Con	−0.74 (0.26)	5.30E−03	−0.53 (0.40)	1.85E−01	1.22 (0.32)	1.35E−04	1.55 (0.41)	2.02E−04
SY‐EXPR	−0.55 (0.37)	1.44E−01	−1.55 (0.81)	5.72E−02	0.96 (0.63)	1.35E−01	0.90 (0.77)	2.44E−01
*Meta‐analysis*	−0.75 (0.11)	**2.54E−12**	−0.85 (0.16)	**1.19E−07**	1.05 (0.15)	**3.54E−12**	1.43 (0.19)	**1.85E−13**
Replication stage (*n* = 456)								
NSCLC−1 cases	−0.23 (0.33)	4.86E−01	−1.35 (0.71)	6.06E−02	0.44 (0.57)	4.46E−01	1.03,(0.53)	5.44E−02
NSCLC−1 controls	−0.85 (0.50)	9.41E−02	−0.61 (0.68)	3.74E−01	1.91 (0.52)	4.15E−04	1.16 (0.54)	3.47E−02
NSCLC−2 cases	−0.98 (0.51)	5.55E−02	−0.83 (0.58)	1.57E−01	1.34 (0.60)	2.63E−02	0.82 (0.69)	2.39E−01
NSCLC−2 controls	−0.80 (0.54)	1.43E−01	−1.98 (0.65)	2.82E−03	0.89 (0.58)	1.29E−01	1.77 (0.77)	2.43E−02
*Meta‐analysis*	−0.57 (0.23)	**1.14E−02**	−1.16 (0.34)	**5.73E−04**	1.18 (0.29)	**4.75E−05**	1.14 (0.31)	**2.62E−04**
*Meta‐analysis of both stages*	−0.72 (0.10)	**1.23E−13**	−0.92 (0.15)	**2.83E−10**	1.07 (0.13)	**1.03E−16**	1.35 (0.16)	**1.61E−17**

The bold values mean the *p* values in the meta‐analysis of discovery stage, validation stage, and both stages, respectively.

Association analyses were performed separately in each sub‐study using linear regression models, with inverse‐normal transformed DNA methylation value included as the dependent variable, natural logarithm transformed BMI as the independent variable, with adjustment for age, gender, smoking status, drinking status, and all surrogate variables.

### 3.3 Correlations of DNA methylation with gene expression levels

To investigate whether the methylation levels of four BMI‐related CpGs were correlated with the expression levels of corresponding genes, we further detected the gene expression profiles in peripheral blood among 144 healthy subjects in the SY‐EXPR study. Eight CpG‐expression probe pairs with qualified expression data were included in the further analysis (Table S4). It was shown that methylation levels at cg11024682 and cg06500161 were significantly inversely associated with the expression levels of *SREBF1* and *ABCG1* transcripts (*p* < 0.05/number of expression probes of the corresponding gene; e.g., on the body of *SREBF1*, β = −7.30, *p *= 3.0E‐03 for the association between cg11024682 and ILMN_1663035; on the body of *ABCG1*, β = −11.76, *p *= 1.9E‐04 for the association between cg06500161 and ILMN_2329927 and β = −10.67, *p *= 7.8E‐04 for the association between cg06500161 and ILMN_1794782). However, we did not find significant associations between methylation levels at cg12593793 and cg17061862 with the expression levels of their corresponding annotated genes *LMNA* and *ZNF143*.

### 3.4 Associations of BMI‐related CpGs and NSCLC risk

**FIGURE 2:**
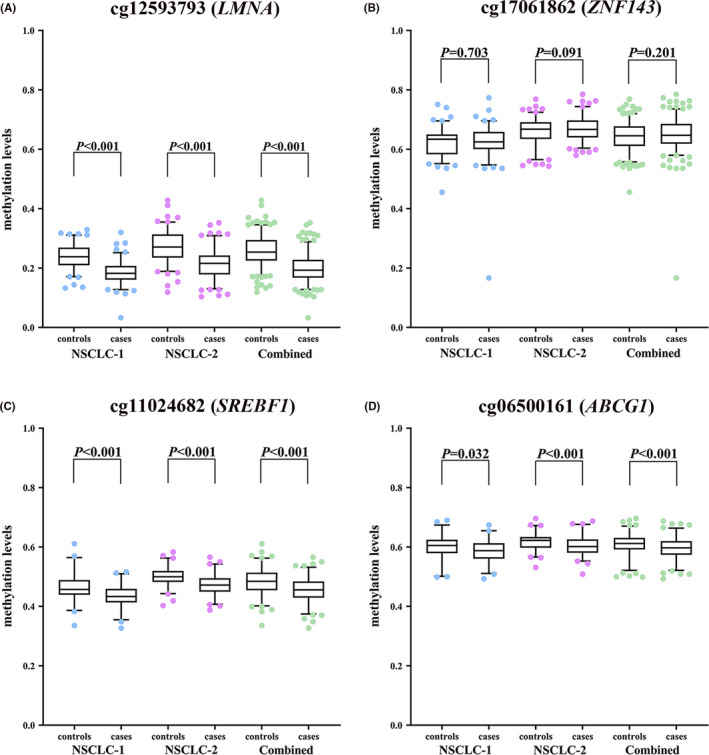
The comparison of the peripheral blood methylation levels of four BMI‐related CpGs between NSCLC cases and controls. (A) cg12593793; (B) cg17061862; (C) cg11024682; (D) cg06500161. NSCLC‐1, the NSCLC case‐control study 1; NSCLC‐2, the NSCLC case‐control study 2

In the combined analysis of two NSCLC case‐control studies, we found that BMI was significantly lower in lung cancer cases than controls [mean ± SD: 23.6 ± 3.0 kg/m^2^ vs. 24.5 ± 3.0 kg/m^2^, *p* < 0.001, data not shown]. Compared with controls, the methylation levels of cg12593793, cg11024682, and cg06500161 were significantly decreased in peripheral blood of lung cancer cases (all *p *< 0.05, Figure 2). However, we did not observe significant differences in the methylation level of cg17061862 between the two populations (*p *= 0.703, 0.091, and 0.201 in NSCLC‐1, NSCLC‐2, and combined studies, respectively).

**FIGURE 3:**
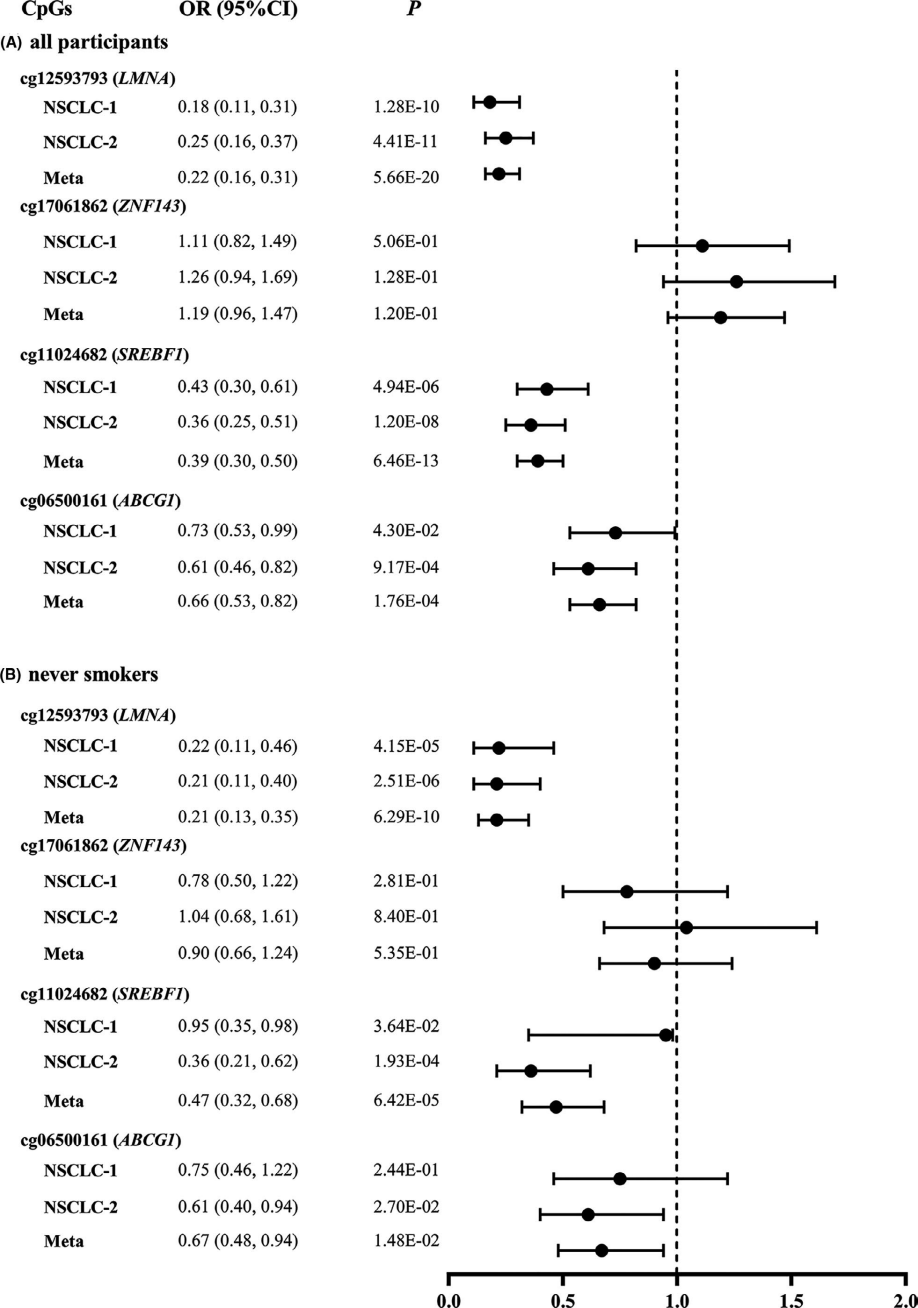
Estimated OR and 95%CI for NSCLC risk per SD increase in DNA methylation levels of four BMI‐related CpGs. (A) All participants; (B) never smokers. SCLC‐1, the NSCLC case‐control study 1; NSCLC‐2, the NSCLC case‐control study 2

The associations between the DNA methylation levels of these four BMI‐related CpGs and NSCLC risk were assessed by logistic regression models in each of the two NSCLC case‐control studies and deriving meta‐analysis ORs and 95%CIs. It was shown that per SD increase in INT transformed methylation β values of cg12593793 in *LMNA*, cg11024682 in *SREBF1*, and cg06500161 in *ABCG1* was associated with a separate 78%, 61%, and 34% decreased risk of lung cancer [meta‐analysis: OR (95%CI) = 0.22 (0.16, 0.31), 0.39 (0.30, 0.50), 0.66 (0.53, 0.82), and *p *= 5.66E‐20, 6.46E‐13, and 1.76E‐04, respectively) (Figure 3A). However, we did not observe the significant associations between the methylation level of cg17061862 in *ZNF143* and lung cancer risk [meta OR (95% CI) = 1.19 (0.96, 1.47), *p *= 1.20E‐01]. Furthermore, no significant heterogeneities were found between the results of two NSCLC case‐control studies (all *p*_heterogeneity_ > 0.05). The sensitivity analysis by further adjustment for major leukocyte counts did not materially change the above associations (Table S5) and the stratification analysis by smoking status (ever vs. never) did not modify the above associations (all *p*_interaction_ > 0.05, Table S6). Among never smokers, the methylation levels of cg12593793, cg11024682, and cg06500161 were also significantly associated with decreased risk of NSCLC [meta‐analysis: OR (95%CI) = 0.21 (0.13, 0.35), 0.47 (0.32, 0.68), 0.67 (0.48, 0.94), and *p* = 6.29E‐10, 6.42E‐05 and 1.48E‐02, respectively] (Figure 3B). In the secondary analysis of the separate associations of these CpGs with risk of LUAD and LUSC, we did not find significant heterogeneity between these two pathological lung cancer subtypes (Figure S3, all *p*_effect‐difference_ > 0.05).

To further testify the possible mediation effects of the above three CpGs, we found that the methylation levels of cg11024682 and cg06500161 could mediate 45.3% and 19.5% of the association between BMI and NSCLC risk, respectively (both *p*_mediation_ < 0.05, Table 3). However, we did not observe the significant mediation effects of cg12593793 on the above association (*p*_mediation_ = 0.315).

**TABLE 3:** Mediation effects of DNA methylation sites on the association between BMI and NSCLC risk

CpG	Total effect		NDE		NIE		Proportion of mediation
	OR (95% CI)	*p*	OR (95% CI)	*p*	OR (95% CI)	*p*	
cg12593793 (*LMNA*)	0.89 (0.82, 0.96)	0.003	0.90 (0.84, 0.97)	0.005	0.98 (0.95, 1.02)	0.315	16.1%
cg11024682 (*SREBF1*)	0.89 (0.83, 0.96)	0.002	0.94 (0.88, 1.01)	0.085	0.95 (0.92, 0.98)	**<0.001**	45.3%
cg06500161 (*ABCG1*)	0.90 (0.84, 0.96)	0.003	0.92 (0.86, 0.98)	0.014	0.98 (0.97, 0.99)	**0.013**	19.5%

Note:

The bold values indicate the significance of mediation effects with *p* values < 0.05.

Methylation beta levels were inverse‐normal transformed, and age, gender, smoking status, and drinking status were adjusted in the models. NDE: natural direct effect of BMI on NSCLC risk. NIE: natural mediation effect by DNA methylation.

## 4 DISCUSSION

In the present study, we identified and replicated four BMI‐related CpGs (cg12593793, cg17061862, cg11024682, and cg06500161) in whole blood through an epigenome‐wide meta‐analysis of BMI in the Chinese populations. The methylation levels of three CpGs (cg12593793, cg11024682, and cg06500161) were inversely associated with NSCLC risk. More importantly, cg11024682 in *SREBF1* and cg06500161 in the *ABCG1* gene could mediate 45.3% and 19.5% of the BMI‐NSCLC association, respectively, suggesting the biological role of DNA methylation on the association between BMI and reduced lung cancer risk in the Chinese populations.

Recently, a pooled analysis of 12 cohort studies with >1.6 million individuals observed that BMI was associated with decreased risk of NSCLC [OR (95% CI) = 0.86 (0.84, 0.89) and 0.94 (95%CI = 0.90, 0.99) for LUAD and LUSC, respectively].^30^ Although the inverse association between BMI and lung cancer risk has been reported, the interpretations remain controversial. Smoking, as the dominant risk factor for lung cancer, could lead to the change of body weight and fat distribution.^31^ Nevertheless, the reported meta‐analysis of published cohort studies confirmed the inverse association in never‐smokers.^8^, ^30^ Another concern is the reverse causation. Subjects who are subsequently diagnosed as lung cancer may show preclinical weight loss. However, the inverse association of BMI and lung cancer remains persisted when excluding the first 5 years or even the first 10 years onset cases in longitudinal cohort studies,^7^, ^30^ providing evidence against reverse causation. Zhou et al conducted a two‐sample multivariable Mendelian randomization (MR) analysis with adjustment for smoking behaviors and found a causal effect of BMI on LUAD [OR (95% CI) = 0.86 (0.77, 0.96), *p* = 0.008]; however, this causal effect of BMI was not observed on LUSC risk [OR (95% CI) = 1.02 (0.96, 1.16), *p* = 0.746].^32^ This multivariable MR analysis takes pleiotropy from BMI and smoking into consideration, which helps to make the relationship between BMI and lung cancer more reliable.

Previous studies evaluating the relationship between BMI and DNA methylation were mainly carried out among European populations.^33^ The CHARGE Consortium published the EWAS of BMI in the European population and found 187 BMI‐related methylation markers,^13^ among which three CpGs (cg12593793, cg11024682, and cg06500161, located in *LMNA*, *SREBF1*, and *ABCG1*, respectively) were replicated in our current study among the Chinese populations, suggesting that BMI‐related DNA methylation showed similar epigenetic changes across different ethnic groups. Consistent with our results, Mendelson et al also observed the associations of BMI with decreased expression of *SREBF1* and *ABCG1* in their study, implying a negative effect of BMI on gene expression via increasing DNA methylation.^12^

The interrelationship between BMI and DNA methylation is complex and possibly bidirectional. Li et al carried out the ICE FALCON (Inference about Causation from Examination of Familial Confounding)^34^ analysis in 479 Australian women from twin families and found that BMI had a causal effect on DNA methylation level at *ABCG1* cg06500161.^35^ Wahl et al calculated a weighted genetic risk score (GRS) based on 29 known BMI SNPs and observed the significant direct effects of BMI‐weighted GRS on methylations of *ABCG1* cg06500161 (*p *= 6.4E‐05) and *SREBF1* cg11024682 (*p *= 4.1E‐03).^13^ Mendelson et al analyzed the data from 2170 individuals and found that methylation levels at 16 CpGs, including cg06500161, were secondary to the difference in BMI.^12^ But they also observed the causal effect of cg11024682 at *SREBF1* on BMI by the reverse MR analysis. Given the cross‐sectional nature of the present EWAS analysis, whether the change in DNA methylation is the cause or consequence of BMI remain unclear. Further bidirectional MR studies are warranted to test the causal relationship.

The epigenetic change in genome‐wide methylation often appears even at the early stage of cancer development.^36^ In our study, the methylation levels of three BMI‐related CpGs (cg12593793, cg11024682, and cg06500161) were observed to be inversely associated with NSCLC risk in whole blood. Although previous studies had found that the methylation level of cg12593793 was lower in current smokers compared with never smokers,^24^, ^37^ we still observed a significantly inverse association of this CpG with NSCLC risk in never smokers. Further stratification analyses suggested that associations of BMI‐related DNA methylation alterations with NSCLC risk were not likely to be confounded by tobacco smoking or histological subtypes, although LUAD and LUSC originated from different cells and had major differences in etiologies and biological patterns.^38^

The mediation effect of DNA methylation is an area of growing interest, in which DNA methylation serves as a vital pathway that connects environmental exposure with health outcomes.^9^ Substantial evidence suggested that DNA methylation might play a crucial role in the associations between BMI and metabolic diseases.^39^, ^40^ In this study, we found that cg11024682 (in *SREBF1*) and cg06500161 (in *ABCG1*) could mediate 45.3% and 19.5% of the association between BMI and decreased lung cancer risk, respectively. *SREBF1* is a vital transcription factor and promotes tumor proliferation, invasion and migration by providing the membrane building materials to support the rapid proliferation of cancer cells.^41^ The *in vitro* assays showed that the inhibition of *SREBF1* increased gefitinib sensitivity in NSCLC cells PC9 and A549.^42^ *ABCG1 *has been shown to be a potential oncogene for lung cancer.^43^ In PC9 and A549, the deficiency of *ABCG1* was reported to inhibit tumor growth by transforming macrophages from a tumor‐promoting phenotype into a tumor‐fighting phenotype.^44^ Interestingly, Wang et al also found that the genetic variants in *ABCG1* (rs225388G>A and rs225390A>G) were associated with survival of lung cancer patients.^43^ The above studies suggested potential functions of *SREBF1* and *ABCG1* in the development of lung cancer, but the etiology underlying the mediation roles of their DNA methylation levels on BMI‐NSCLC association still needs to be explored by further investigations.

To our knowledge, this is the largest study to conduct the EWAS of BMI in the Chinese populations. This study is also the first to provide epidemiological evidence on the mediation role of DNA methylation in the effect of BMI on NSCLC, which may help to provide insights into potential mechanisms. In the data analysis, we used stringent conditions for QC and a variety of statistical methods (e.g., SVA method, sensitivity analysis, and stratification analysis) to ensure reliability. SVA method is an appropriate correction method to reduce the confounding bias introduced by cell type distributions in the associations of BMI or lung cancer with DNA methylation in the whole blood.^45^ However, some limitations should be noted. First, we used populations from different areas of China to perform the EWAS of BMI; the heterogeneity of these populations may exist. However, meta‐analysis of the subset populations showed no heterogeneity for validated BMI‐related CpGs. In addition, three of the four identified BMI‐related methylation loci were previously reported among European populations, suggesting that the current results are reliable.^12^ Second, the case‐control study design of lung cancer is unable to establish a causal relationship between DNA methylation and lung cancer risk. Reverse causal relationship may exist, given the blood samples of NSCLC patients taken before surgical operation. Further longitudinal cohort study and more bidirectional MR analyses are needed to disclosure the causal associations and biological functions. Third, although we adjusted smoking status in the association analysis between DNA methylation and lung cancer, and similar pattern of results were shown both in never smokers and the overall population, the possibility of residual confounding may still remain. Fourth, another concern when conducting EWAS in peripheral blood is whether blood‐derived DNA methylation changes can also occur in target tissues. However, cross‐tissue studies have found the moderate‐to‐high consistency between blood and adipose tissue in BMI‐DNA methylation associations.^13^, ^46^ From analyses of 237 non‐tumor lung tissues, Stueve et al found that EWAS analysis of DNA methylation in lung tissue showed concordance with blood studies.^47^ In addition to DNA methylation, the association of BMI and lung cancer may also be mediated by other biological mechanisms, such as oxidative stress and DNA adducts damage.^48^ Further studies are required to validate the current findings and explore other possible biological functions.

In conclusion, we found novel and reproducible associations between BMI and blood methylation levels at four CpGs (cg12593793, cg17061862, cg11024682, and cg06500161) in the Chinese populations. In addition, the DNA methylations of cg11024682 in *SREBF1* and cg06500161 in *ABCG1* could mediate about a quintile‐to‐half of the association between BMI and reduced NSCLC risk. Although our findings require further confirmation, findings in this study may gain insights into the epigenetic regulations underlying obesity and reveal potential epigenetic targets for lung cancer prevention.

## CONFLICT OF INTEREST

The authors declared no conflict of interest.

## ETHICS APPROVAL AND CONSENT TO PARTICIPATE

All participants were provided informed consent and this work has received approval for research ethics from the Ethics Committee of Tongji Medical College, Huazhong University of Science and Technology. A proof/certificate of approval is available upon request (no. S335).

## Supporting information

Supplementary Material

## Data Availability

The datasets used and/or analyzed during the current study are available from the corresponding author on reasonable request.
