# Supplementary material for: DNA methylome analysis identifies BMI‐related epigenetic changes associated with non‐small cell lung cancer susceptibility
Source: Cancer Med. 2021 May 3;10(11):3770–81. doi: 10.1002/cam4.3906 (PMC8178488; doi:10.1002/cam4.3906)
Supplement: Supplementary file 1 — Supplementary Material [file CAM4-10-3770-s001.docx]

**Supplementary materials**

**Study population**

In the main EWAS meta-analysis we explored the association of DNA methylation with BMI using data from a total of 2 266 participants, within 1 810 subjects in the discovery stage and 456 subjects in the replication stage. Details of participants in discovery stage have been described in previous studies^1, 2^. The information of all participants is mentioned briefly below.

***The COW panel*.** The COW study is a prospective occupational cohort study established in 2010 in a coke-oven plant in in Wuhan City, Hubei Province, China^3^. A total of 1 628 workers were recruited at baseline. We randomly selected 144 workers who met the following criteria: 1) provided blood and urine samples; 2) had worked in the same plant for more than five years; 3) had total urinary monohydroxy polycyclic aromatic hydrocarbons (OH-PAHs) in the high tertile at baseline; 4) had no self-reported diseases or discomfort; 5) had no febrile illness or infectious conditions in the two weeks before the time of blood collection, and did not take any prescribed medication in a month before the time of blood collection; 6) had a body mass index (BMI) of 18.0-30.5 kg/m^2^. After quality control (QC) of DNA methylation data, the final number of subjects (abbreviated as COW-WH) came to 137.

***The Acute Coronary syndrome (ACS)-patients panel*.** 206 acute coronary syndrome (ACS) patients were recruited, with 103 patients from Wuhan Union hospital and Wuhan Central hospital in Hubei Province, China, and another 103 patients from Bao’an Hospital and People’s Hospital of Zhuhai in Guangdong Province, China. The diagnosis of ACS, defined as unstable angina pectoris, acute non-ST-elevation myocardial infarction and acute ST-elevation myocardial infarction, was confirmed by expert physicians reviewing on medical records, symptoms, coronary angiography and electrocardiogram. Patients, who simultaneously suffered from complications, including congenital heart disease, cardiomyopathy, autoimmune diseases, infectious diseases, chronic obstructive pulmonary disease, tuberculosis, liver diseases, kidney diseases or malignant tumors were excluded. After QC, 101 patients from Wuhan (abbreviated as ACS-WH) and 97 patients from Guangdong (abbreviated as ACS-GD) were included.

***The WHZH Cohort panel*.** The WHZH cohort study is a prospective, community-based cohort study, recruiting 3 053 residents in Wuhan and 1 759 residents in Zhuhai at baseline in 2011^4^. Individuals were all 18-80 years old and had lived in the same community for more than 5 years. After excluding individuals who 1) reported severe illness at baseline interview or showed abnormalities at baseline examination; 2) had the fever or infectious illness within two weeks before blood drawn; 3) took prescribed medicine in the past month; 4) did not donate blood and urine samples at baseline, 180 Wuhan residents were selected as healthy controls to match ACS-WH patients (matched for age, gender and BMI, n=103) and/or to match COWs (matched for age, gender and BMI, and with total urinary OH-PAHs in the low tertile, n=144; ACS-WH patients and COWs shared 64 controls). Another 103 residents in Guangdong Province were selected to match the above ACS-GD patients (matched for age, gender and BMI). After QC, we included 162 residents from Wuhan (abbreviated as **WHZH-WH**) and 99 residents from Guangdong Province (abbreviated as **WHZH-ZH**) in the present analysis.

***The DFTJ panel for incident ACS*.** Established in 2008, the DFTJ cohort study is a prospective cohort study based on retired workers from a Motor Corporation in Shiyan City, Hubei Province, China^5^. 27 009 participants were recruited to complete baseline questionnaires and medical examinations. At the first follow-up in 2013, 96.2% of the participants were successfully followed-up. And the incidents of coronary vascular disease (CVD), diabetes and cancer were registered up to December 2013.

The ACS-control panel is a nested case-control study based on the DFTJ cohort study. The definition and diagnosis of ACS were consistent with the described above. Subjects eligible for the nested case-control study were the ones who 1) did not report coronary heart disease (CHD), stroke or cancer at baseline; 2) were successfully followed-up; 3) donated blood samples at baseline. Until the end of 2013, we identified 1 146 new incident ACS cases. Among them, 344 incident ACS cases (abbreviated as **DFTJ-ACS**) were selected at random. The 1:1 age (±3 years), gender and sampling time at baseline (±40 days) frequency-matched 344 healthy controls (abbreviated as **DFTJ-Con**). All participants passed QC criteria.

***The Shanghai Women’s and Men’s health study (SWMHS) panel*.** SWMHS is an ongoing population-based prospective cohort study based on general population^6^. 74 941 women aged from 40 to 70 years old were recruited during 1996 to 2000, and 61 491 men aged from 40 to 74 years old were recruited from 2002 to 2006^6, 7^. At baseline, all participants received a face-to-face interview and a physical examination. Follow-ups were performed at two-years intervals and the information of characteristics, lifestyles, diets or diseases were collected.

We conducted a nested case-control study based on the SWMHS cohort study. Subjects eligible for the nested case-control study were the ones who provided biospecimens and reported no history of CHD, stroke or cancer at baseline. Cases were defined as subjects with incident CHD, that occurred after the baseline survey and before the end of 2009. During the 10-year follow-up period, 1 095 CHD patients were identified. After excluding cases who had no urine samples or suffered from CVD, stroke or cancer at baseline, 191 ACS cases were selected (abbreviated as **SWMHS-ACS**). 191 controls, who were free of CVD, stroke and cancer at baseline and during the follow-up period, were matched to cases by age, gender, sampling time at baseline and the use of antibiotics in the past week (abbreviated as **SWMHS-Con**).

***The SY-EXPR panel*.** To investigate the effects of DNA methylation on gene expression, 144 healthy subjects were recruited in at the Department of Health Examination in Sinopharm Dongfeng General Hospital in Shiyan City, Hubei Province, China, during April and May of 2015. The selected participants met the following criteria: 1) aged from 20 to 70 years old; 2) reported no diseases or discomfort; 3) reported no fever or infectious diseases in 2 weeks before the time of baseline examination and did not take prescribed medicine in a month before the time of baseline examination; 4) donated blood and urine samples. All 144 subjects passed QC and were included in the final analyses.

***The*** ***Lung Cancer panel*.** The validation stage consisted of two non-small cell lung cancer (NSCLC) case-control studies (abbreviated as NSCLC-1 and NSCLC-2). The first study included 109 pairs of lung cancer cases and controls, matched at age (±2 years) and gender; the second study included 126 pairs of lung cancer cases and controls, also matched at age (±2 years) and gender. NSCLC cases of NSCLC-1 and NSCLC-2 underwent surgical resection of primary lung cancer during June 2012 to December 2014 and during June 2018 to December 2019, respectively, in the Department of Thoracic Surgery, Tongji Hospital in Wuhan City, Hubei Province, China. Patients who 1) had been treated with preoperative chemoradiotherapy, and biotherapy; and 2) suffered from multiple lung diseases simultaneously, such as tuberculosis, pneumonia and silicosis were excluded. Information on demographic characteristics, lifestyles and medical history were obtained through hospital medical records and face-to-face interviews. Further, the peripheral blood samples of lung cancer patients were collected before surgical operation. The diagnosis of NSCLC was based on pathological examination from surgical pathology archives. Healthy controls, who were free of malignant cancer, diabetes, stroke and CVD, were 1:1 frequency matched with cases at recruitment for age and gender from the physical examination center, Tongji Hospital, during the same periods with patients. After QC, 105 cases [including 54 of lung adenocarcinoma (LUAD) and 51 of lung squamous carcinoma (LUSC)] and 103 controls were retained in NSCLC-1, and 125 cases (including 78 of LUAD and 47 of LUSC) and 123 controls were kept in NSCLC-2.

**Laboratory Assays**

***Genome-wide DNA methylation*.** Genomic DNA samples were extracted from peripheral blood by using the BioTeke Whole Blood DNA Extraction Kit (BioTeke, Beijing, China) and were stored at -80℃ until laboratory testing. DNA concentration was quantitated with the NanoDrop spectrophotometer (Thermo Scientific, Wilmington, DE, USA). 1000 ng genomic DNA was bisulfite converted with the EZ DNA Methylation kit (Zymo research, Orange, CA, USA) according to manufacturers’ instructions. Methylation of genomic DNA was quantified by using the Illumina Human Methylation 450K array (Illumina, Inc., Boston, USA) for participants in the discovery stage as well as NSCLC-1 study in the validation stage or Illumina Human Methylation EPIC array for participants in the NSCLC-2 study in the validation stage.

Raw signal intensities were retrieved from the IDAT files using the function readIDAT of the R package “minfi”, followed by background correction with the function bgcorrect.illumina^8^. Multidimensional Scaling (MDS) analysis was applied to determine signal outliers. QC included removal of CpGs if they: (1) had a missing rate >20% across samples (missing means a probe with detection *P*>0.01 or bead counts<3); (2) located on sex chromosome; (3) assayed single nucleotide polymorphisms (SNPs) rather than CpGs (65 SNP-probes on 450K array and 59 SNP-probes on EPIC array); (4) had been identified as cross-hybridizing with other genomic locations; (5) potentially contained or extended on SNPs with MAF>0.05 in the 1000 Genomes Project for ASN population (4 and 5 contained totally 41 296 probes ^9^), as well as samples if they: (1) were MDS outliers; (2) had a missing rate>0.05 across probes; (3) were with individual-call rate <0.98; (4) had incorrect sex match. After filtering, methylation values at 430 302 CpGs were normalized by Dasen implemented in R package “wateRmelon” in the discovery stage, and in the validation stage, methylation values at 433 439 and 808 064 CpGs in NSCLC-1 and NSCLC-2, respectively, were normalized by functional normalization of the “minfi” package ^8, 10^. The β value, which represented the ratio of the intensity of the methylated-probe signal to the total locus signal intensity, was used in the further analyses.

***Gene expression.*** For the 144 participants recruited in SY-EXPR study, total RNA was isolated within two hours after blood collection using TRIZOL LS solution (Invitrogen, USA). Gene expression profiles in total RNA were detected by using HumanHT-12 v4 BeadChip (Illumina). Detailed information of the laboratory and QC methods had been published previously^2^. Briefly, raw expression values were subtracted by GenomeStudio (Illumina, USA) and all unexpressed signals (<0) were assigned as 0. Expression data were normalized using quantile-quantile normalization within the “beadarray” package in R^11^. All samples passed QC and provided qualified data for analysis.

**References**

1. Li J, Zhu X, Yu K, Jiang H, Zhang Y, Deng S *et al.* Genome-Wide Analysis of DNA Methylation and Acute Coronary Syndrome. *Circ Res* 2017; **120**(11)**:** 1754-1767.

2. Zhu X, Li J, Deng S, Yu K, Liu X, Deng Q *et al.* Genome-Wide Analysis of DNA Methylation and Cigarette Smoking in a Chinese Population. *Environ Health Perspect* 2016; **124**(7)**:** 966-973.

3. Li X, Feng Y, Deng H, Zhang W, Kuang D, Deng Q *et al.* The dose-response decrease in heart rate variability: any association with the metabolites of polycyclic aromatic hydrocarbons in coke oven workers? *PLoS One* 2012; **7**(9)**:** e44562.

4. Song Y, Hou J, Huang X, Zhang X, Tan A, Rong Y *et al.* The Wuhan-Zhuhai (WHZH) cohort study of environmental air particulate matter and the pathogenesis of cardiopulmonary diseases: study design, methods and baseline characteristics of the cohort. *BMC Public Health* 2014; **14:** 994.

5. Wang F, Zhu J, Yao P, Li X, He M, Liu Y *et al.* Cohort Profile: the Dongfeng-Tongji cohort study of retired workers. *Int J Epidemiol* 2013; **42**(3)**:** 731-740.

6. Zheng W, Chow WH, Yang G, Jin F, Rothman N, Blair A *et al.* The Shanghai Women's Health Study: rationale, study design, and baseline characteristics. *Am J Epidemiol* 2005; **162**(11)**:** 1123-1131.

7. Cai H, Zheng W, Xiang YB, Xu WH, Yang G, Li H *et al.* Dietary patterns and their correlates among middle-aged and elderly Chinese men: a report from the Shanghai Men's Health Study. *Br J Nutr* 2007; **98**(5)**:** 1006-1013.

8. Aryee MJ, Jaffe AE, Corrada-Bravo H, Ladd-Acosta C, Feinberg AP, Hansen KD *et al.* Minfi: a flexible and comprehensive Bioconductor package for the analysis of Infinium DNA methylation microarrays. *Bioinformatics* 2014; **30**(10)**:** 1363-1369.

9. Chen YA, Lemire M, Choufani S, Butcher DT, Grafodatskaya D, Zanke BW *et al.* Discovery of cross-reactive probes and polymorphic CpGs in the Illumina Infinium HumanMethylation450 microarray. *Epigenetics* 2013; **8**(2)**:** 203-209.

10. Pidsley R, CC YW, Volta M, Lunnon K, Mill J, Schalkwyk LC. A data-driven approach to preprocessing Illumina 450K methylation array data. *BMC Genomics* 2013; **14:** 293.

11. Dunning MJ, Smith ML, Ritchie ME, Tavare S. beadarray: R classes and methods for Illumina bead-based data. *Bioinformatics* 2007; **23**(16)**:** 2183-2184.

**Table of Contents**

**Table S1.** The 20 CpGs associated with BMI at false discovery rate (FDR<0.05) in the discovery stage.

**Table S2.** The associations between 20 CpGs and BMI in the replication stage, with 4 CpGs reached the significant level at *P*<0.05.

**Table S3.** The associations between BMI and 20 CpGs among never smokers in the discovery stage, replication stage and the meta-analysis of both stages.

**Table S4.** Methylation-gene expression correlations of the 4 BMI-related CpGs in SY-EXPR population (n=144).

**Table S5.** Estimated OR and 95%CI for NSCLC risk per SD increase in DNA methylation levels of 4 BMI-related CpGs after adjustment for major leukocyte counts.

**Table S6.** Estimated OR and 95%CI for NSCLC risk per SD increase in the methylation levels of 4 BMI-related CpGs after stratified by the smoking status.

**Figure S1.** Epigenome-wide meta-analysis of BMI in the discovery stage.

(A) Manhattan plot, all points above the solid line are at FDR<0.05 (20 CpGs).

(B) Quantile-quantile plot of the EWAS results.

**Note:** a fixed-effect meta-analysis of EWAS weighted on the inverse variance was performed to establish the observational associations between BMI and differential DNA methylations in the discovery stage.

**Figure S2.** Associations of all methylation sites (±600 kbps) surrounding the 4 BMI-related CpGs with BMI.

**Note:** This plot was depicted based on the meta-analysis of both discovery stage and validation stage. The horizontal axis denotes the position of gene, and the vertical one denotes the value of -log_10_*P*. The diamond nodes represented the discovered and replicated 4 CpGs, and the dots represented the methylation sites (±600 kbps) surrounding the 4 CpGs.

**Figure S3.** Comparison of effect sizes between lung adenocarcinoma (LUAD) and lung squamous carcinoma (LUSC) for the three CpGs (cg12593793, cg11024682 and cg06500161) related to both BMI and lung cancer risk in NSCLC-1 and NSCLC-2 subsets.

**Note:** Black bars stand for effect sizes of LUAD and gray bars correspond to effect sizes of LUSC.

| **Table S1. The 20 CpGs associated with BMI at false discovery rate (FDR<0.05) in the discovery stage.** | | | | | | | | | | | | | | | | | | | | | |
| --- | --- | --- | --- | --- | --- | --- | --- | --- | --- | --- | --- | --- | --- | --- | --- | --- | --- | --- | --- | --- | --- |
| **Sub-study** | | **cg03418289** | **cg12593793** | **cg24674445** | **cg25570328** | **cg06398474** | **cg16984944** | **cg01741041** | **cg07914621** | **cg01866330** | **cg25392060** | **cg17061862** | **cg00574958** | **cg00862597** | **cg02484673** | **cg23415756** | **cg11024682** | **cg23985214** | **cg23510258** | **cg07769588** | **cg06500161** |
| **ACS-WH** | **β** | -0.06 | 0.04 | -2.87 | -0.83 | -1.46 | -1.85 | -0.28 | -1.50 | 1.67 | -0.22 | -0.42 | -0.09 | 0.38 | -0.41 | -2.59 | 1.10 | 2.03 | 1.92 | 1.10 | 1.20 |
|  | ***P*** | 9.39E-01 | 9.30E-01 | 5.99E-04 | 1.16E-01 | 2.41E-02 | 4.35E-03 | 6.28E-01 | 4.97E-02 | 5.89E-02 | 7.52E-01 | 5.22E-01 | 9.18E-01 | 5.41E-01 | 4.65E-01 | 4.14E-03 | 1.71E-01 | 1.25E-02 | 6.48E-04 | 1.59E-01 | 2.15E-01 |
| **ACS-GD** | **β** | 0.74 | -0.45 | -0.45 | -0.46 | 0.86 | -1.39 | -0.27 | -1.99 | 3.55 | -0.22 | -1.13 | -0.66 | -0.18 | -0.72 | -0.74 | -0.41 | 0.66 | -0.01 | 1.12 | -0.25 |
|  | ***P*** | 2.80E-01 | 3.64E-01 | 6.32E-01 | 3.73E-01 | 2.63E-01 | 5.74E-02 | 6.25E-01 | 3.07E-03 | 6.48E-05 | 7.74E-01 | 1.23E-01 | 4.11E-01 | 8.10E-01 | 1.54E-01 | 4.64E-01 | 6.58E-01 | 4.88E-01 | 9.86E-01 | 2.51E-01 | 8.03E-01 |
| **WHZH-WH** | **β** | 0.24 | -0.24 | -0.96 | -0.86 | -0.91 | -1.20 | 0.25 | -0.73 | 1.74 | 1.24 | 0.34 | -1.43 | 0.58 | -0.53 | -1.38 | 1.73 | 0.56 | 0.82 | 1.00 | 1.97 |
|  | ***P*** | 6.66E-01 | 4.32E-01 | 1.16E-01 | 5.33E-03 | 2.37E-02 | 1.36E-02 | 4.47E-01 | 8.26E-02 | 1.08E-02 | 1.22E-02 | 4.21E-01 | 1.10E-02 | 1.73E-01 | 1.19E-01 | 2.54E-02 | 2.05E-04 | 2.65E-01 | 3.75E-02 | 6.99E-02 | 2.73E-03 |
| **WHZH-ZH** | **β** | 0.79 | -1.28 | 0.45 | -0.10 | 0.02 | -0.29 | 0.49 | -0.78 | 1.82 | -0.08 | -0.54 | -1.40 | 0.59 | -0.23 | 0.10 | 1.16 | 1.45 | -0.36 | 0.48 | 0.85 |
|  | ***P*** | 3.48E-01 | 9.72E-03 | 6.76E-01 | 8.27E-01 | 9.79E-01 | 6.92E-01 | 4.04E-01 | 3.29E-01 | 8.39E-02 | 9.16E-01 | 4.74E-01 | 1.22E-01 | 4.16E-01 | 6.77E-01 | 9.20E-01 | 1.09E-01 | 1.64E-01 | 5.95E-01 | 6.02E-01 | 4.02E-01 |
| **COW-WH** | **β** | 0.30 | -0.81 | -0.25 | 0.15 | -1.03 | -0.16 | 0.71 | -0.65 | 1.88 | 0.73 | -1.77 | -1.63 | 0.54 | -0.50 | 0.88 | 1.69 | 1.25 | 1.14 | 0.52 | 0.82 |
|  | ***P*** | 6.13E-01 | 7.14E-02 | 7.21E-01 | 7.07E-01 | 3.42E-02 | 7.94E-01 | 6.94E-02 | 2.71E-01 | 1.60E-02 | 2.53E-01 | 4.44E-04 | 2.34E-02 | 2.75E-01 | 2.62E-01 | 2.13E-01 | 4.22E-03 | 3.05E-02 | 2.95E-02 | 4.42E-01 | 2.41E-01 |
| **SWMHS-ACS** | **β** | 1.32 | -1.32 | -0.31 | -0.33 | -0.42 | -1.26 | 1.02 | -0.80 | -0.24 | 0.44 | -0.96 | -0.34 | 1.05 | -0.54 | -2.06 | 1.15 | 1.23 | 0.43 | 1.07 | 1.60 |
|  | ***P*** | 1.56E-02 | 4.89E-04 | 5.91E-01 | 2.67E-01 | 3.34E-01 | 7.94E-03 | 2.72E-02 | 1.12E-01 | 6.81E-01 | 3.27E-01 | 8.68E-02 | 5.31E-01 | 1.08E-02 | 1.89E-01 | 5.59E-05 | 1.11E-02 | 3.27E-02 | 2.73E-01 | 1.86E-02 | 5.67E-03 |
| **SWMHS-Con** | **β** | 1.90 | -0.66 | -1.72 | 0.09 | -0.67 | -0.37 | 1.01 | -0.73 | 0.97 | 0.80 | -1.44 | -1.24 | 0.83 | -0.72 | -0.64 | 0.94 | 0.66 | 0.86 | 0.88 | 1.17 |
|  | ***P*** | 1.28E-04 | 2.83E-02 | 4.95E-04 | 7.29E-01 | 8.00E-02 | 3.14E-01 | 7.21E-03 | 1.03E-01 | 6.67E-02 | 5.74E-02 | 3.14E-03 | 9.03E-03 | 2.00E-02 | 8.50E-02 | 2.01E-01 | 1.66E-02 | 1.72E-01 | 5.37E-03 | 5.49E-02 | 1.88E-02 |
| **DFTJ-ACS** | **β** | 0.45 | -1.18 | -0.55 | -0.93 | -1.09 | -0.57 | 0.40 | -0.55 | 0.54 | 1.25 | -1.15 | -1.01 | 0.77 | -0.91 | -1.25 | 0.55 | 0.73 | 0.54 | 0.28 | 1.92 |
|  | ***P*** | 2.73E-01 | 4.24E-06 | 1.52E-01 | 6.71E-05 | 2.20E-04 | 7.59E-02 | 1.86E-01 | 1.38E-01 | 1.98E-01 | 6.30E-04 | 3.76E-03 | 1.51E-02 | 1.03E-02 | 1.44E-03 | 3.78E-03 | 7.67E-02 | 4.93E-02 | 2.92E-02 | 3.83E-01 | 1.47E-06 |
| **DFTJ-Con** | **β** | 1.02 | -0.74 | -0.90 | -0.82 | -0.41 | -0.36 | 0.90 | -0.72 | -0.17 | 1.03 | -0.53 | -0.61 | 0.87 | -0.35 | -0.76 | 1.22 | 0.50 | 0.32 | 0.87 | 1.55 |
|  | ***P*** | 1.18E-02 | 5.30E-03 | 2.78E-02 | 2.41E-03 | 2.10E-01 | 2.58E-01 | 3.21E-03 | 7.57E-02 | 7.00E-01 | 7.39E-03 | 1.85E-01 | 1.50E-01 | 2.94E-03 | 2.25E-01 | 7.56E-02 | 1.35E-04 | 2.10E-01 | 2.34E-01 | 9.96E-03 | 2.02E-04 |
| **SY-EXPR** | **β** | 0.99 | -0.55 | -1.24 | -0.44 | -0.43 | -0.49 | 1.89 | -0.72 | 2.54 | 1.31 | -1.55 | -0.42 | 0.61 | -0.95 | 0.43 | 0.96 | 0.86 | 0.49 | 1.04 | 0.90 |
|  | ***P*** | 1.39E-01 | 1.44E-01 | 1.10E-01 | 2.71E-01 | 4.87E-01 | 3.65E-01 | 2.57E-04 | 3.43E-01 | 2.87E-03 | 4.44E-02 | 5.72E-02 | 5.89E-01 | 2.70E-01 | 6.98E-02 | 5.69E-01 | 1.35E-01 | 2.10E-01 | 2.17E-01 | 1.46E-01 | 2.44E-01 |
| ***Meta-analysis*** | |  |  |  |  |  |  |  |  |  |  |  |  |  |  |  |  |  |  |  |  |
|  | **β** | 0.84 | -0.75 | -0.90 | -0.51 | -0.71 | -0.68 | 0.65 | -0.81 | 0.92 | 0.82 | -0.85 | -0.91 | 0.73 | -0.60 | -0.94 | 1.05 | 0.84 | 0.61 | 0.75 | 1.43 |
|  | **se** | 0.17 | 0.11 | 0.18 | 0.1 | 0.14 | 0.14 | 0.13 | 0.16 | 0.19 | 0.16 | 0.16 | 0.18 | 0.13 | 0.12 | 0.19 | 0.15 | 0.17 | 0.12 | 0.16 | 0.19 |
|  | ***P*** | 2.14E-06 | 2.54E-12 | 2.22E-06 | 6.37E-07 | 1.93E-06 | 2.32E-06 | 2.26E-07 | 8.13E-07 | 9.55E-07 | 4.16E-07 | 1.19E-07 | 1.21E-06 | 1.18E-07 | 1.66E-06 | 2.12E-06 | 3.54E-12 | 9.66E-07 | 6.55E-07 | 1.81E-06 | 1.85E-13 |
|  | **FDR** | **4.99E-02** | **5.07E-07** | **4.99E-02** | **3.13E-02** | **4.99E-02** | **4.99E-02** | **1.62E-02** | **3.46E-02** | **3.46E-02** | **2.56E-02** | **1.03E-02** | **4.01E-02** | **1.03E-02** | **4.99E-02** | **4.99E-02** | **5.07E-07** | **3.46E-02** | **3.13E-02** | **4.99E-02** | **7.97E-08** |
| **Note:** Association analyses were performed separately in each sub-study using linear regression models, with inverse-normal transformed methylation beta values as dependent variables, natural logarithm transformed BMI as the independent variable, and adjustment for age, gender, smoking status, drinking status, and all surrogate variables (SV). Results from each sub-study were combined using fixed-effect meta-analysis. | | | | | | | | | | | | | | | | | | | | | |

| **Table S2. The associations between 20 CpGs and BMI in the replication stage, with 4 CpGs reached the significant level at *P*<0.05.** | | | | | | | | | | | | | | | | | | |
| --- | --- | --- | --- | --- | --- | --- | --- | --- | --- | --- | --- | --- | --- | --- | --- | --- | --- | --- |
| **CpGs** | **Chr.** | **Position** | **Nearest Gene** | **NSCLC-1 cases** | |  | **NSCLC-1 controls** | |  | **NSCLC-2 cases** | |  | **NSCLC-2 controls** | |  | **Meta-analysis** | | |
|  |  |  |  | **β** | ***P*** |  | **β** | ***P*** |  | **β** | ***P*** |  | **β** | ***P*** |  | **β** | **se** | ***P*** |
| cg03418289 | 1 | 145477137 | *LIX1L* | 0.43 | 3.98E-01 |  | 0.23 | 6.01E-01 |  | 0.23 | 6.70E-01 |  | 0.71 | 2.56E-01 |  | 0.36 | 0.26 | 1.76E-01 |
| **cg12593793** | **1** | **156074135** | ***LMNA*** | **-0.23** | **4.86E-01** |  | **-0.85** | **9.41E-02** |  | **-0.98** | **5.55E-02** |  | **-0.80** | **1.43E-01** |  | **-0.57** | **0.23** | **1.14E-02** |
| cg24674445 | 1 | 156076363 | *LMNA* | 0.00 | 9.97E-01 |  | -0.07 | 9.26E-01 |  | 1.66 | 2.32E-02 |  | -0.23 | 7.78E-01 |  | 0.30 | 0.36 | 3.98E-01 |
| cg25570328 | 2 | 108903952 | *SULT1C2* | -0.39 | 3.14E-01 |  | -0.26 | 4.46E-01 |  | -0.88 | 3.84E-02 |  | 0.09 | 8.47E-01 |  | -0.36 | 0.20 | 7.39E-02 |
| cg06398474 | 2 | 240291509 | *HDAC4* | -0.95 | 7.41E-02 |  | 0.08 | 8.88E-01 |  | NA | NA |  | NA | NA |  | NA | NA | NA |
| cg16984944 | 3 | 99979425 | *TBC1D23* | -0.05 | 9.00E-01 |  | -0.10 | 8.00E-01 |  | 0.07 | 8.23E-01 |  | -0.24 | 6.34E-01 |  | -0.05 | 0.20 | 8.12E-01 |
| cg01741041 | 7 | 5567862 | *ACTB* | 0.07 | 8.73E-01 |  | 0.33 | 4.83E-01 |  | -0.14 | 7.94E-01 |  | -0.46 | 4.85E-01 |  | 0.04 | 0.26 | 8.91E-01 |
| cg07914621 | 7 | 150101498 | *LOC728743* | -0.45 | 4.21E-01 |  | -0.23 | 7.18E-01 |  | -0.45 | 4.95E-01 |  | 1.23 | 1.05E-01 |  | -0.11 | 0.33 | 7.29E-01 |
| cg01866330 | 8 | 142235430 | *SLC45A4* | 0.10 | 8.69E-01 |  | -0.52 | 4.22E-01 |  | -1.06 | 6.01E-02 |  | 0.65 | 3.10E-01 |  | -0.26 | 0.32 | 4.18E-01 |
| cg25392060 | 8 | 142297121 | *SLC45A4* | -0.20 | 7.55E-01 |  | 0.77 | 2.60E-01 |  | -1.54 | 1.48E-02 |  | 1.87 | 4.44E-03 |  | 0.18 | 0.33 | 5.84E-01 |
| **cg17061862** | **11** | **9590431** | ***ZNF143*** | **-1.35** | **6.06E-02** |  | **-0.61** | **3.74E-01** |  | **-0.83** | **1.57E-01** |  | **-1.98** | **2.82E-03** |  | **-1.16** | **0.34** | **5.73E-04** |
| cg00574958 | 11 | 68607622 | *CPT1A* | 0.81 | 1.76E-01 |  | -1.21 | 5.99E-02 |  | -1.49 | 9.21E-03 |  | 0.01 | 9.92E-01 |  | -0.49 | 0.32 | 1.24E-01 |
| cg00862597 | 14 | 61750802 | *PRKCH* | -0.20 | 6.79E-01 |  | 0.45 | 3.15E-01 |  | 0.37 | 4.22E-01 |  | -0.14 | 7.75E-01 |  | 0.14 | 0.24 | 5.62E-01 |
| cg02484673 | 16 | 87674713 | *JPH3* | -0.03 | 9.64E-01 |  | 0.11 | 8.03E-01 |  | 0.54 | 3.03E-01 |  | -0.14 | 7.88E-01 |  | 0.12 | 0.26 | 6.49E-01 |
| cg23415756 | 17 | 8925752 | *NTN1* | 0.12 | 8.12E-01 |  | -0.01 | 9.82E-01 |  | 0.16 | 7.79E-01 |  | -0.29 | 6.42E-01 |  | 0.02 | 0.27 | 9.51E-01 |
| **cg11024682** | **17** | **17730094** | ***SREBF1*** | **0.44** | **4.46E-01** |  | **1.91** | **4.15E-04** |  | **1.34** | **2.63E-02** |  | **0.89** | **1.29E-01** |  | **1.18** | **0.29** | **4.75E-05** |
| cg23985214 | 17 | 76356261 | *SOCS3* | 0.00 | 9.95E-01 |  | -0.05 | 9.23E-01 |  | 0.16 | 7.65E-01 |  | -0.42 | 5.24E-01 |  | -0.04 | 0.28 | 8.75E-01 |
| cg23510258 | 17 | 80840821 | *TBCD* | -0.88 | 1.26E-01 |  | 1.19 | 1.74E-02 |  | 0.18 | 6.83E-01 |  | 0.53 | 3.75E-01 |  | 0.31 | 0.27 | 2.48E-01 |
| cg07769588 | 19 | 10655622 | *ATG4D* | 0.35 | 6.02E-01 |  | 0.85 | 2.49E-01 |  | 0.17 | 7.67E-01 |  | -0.07 | 9.19E-01 |  | 0.31 | 0.34 | 3.59E-01 |
| **cg06500161** | **21** | **43656587** | ***ABCG1*** | **1.03** | **5.44E-02** |  | **1.16** | **3.47E-02** |  | **0.82** | **2.39E-01** |  | **1.77** | **2.43E-02** |  | **1.14** | **0.31** | **2.62E-04** |
| **Note:** Association analyses were performed separately in each sub-study using linear regression models, with inverse-normal transformed methylation levels as dependent variables, ln-transformed BMI as the independent variable, with adjustment for age, gender, smoking status, drinking status, and all SVs. Results from each sub-study were combined using fixed-effect meta-analysis. | | | | | | | | | | | | | | | | | | |

| **Table S3. The associations between BMI and 20 CpGs among never smokers in the discovery stage, replication stage, and the meta-analysis of both stages.** | | | | | | | | | |
| --- | --- | --- | --- | --- | --- | --- | --- | --- | --- |
| **CpGs** |  | **Discovery stage** | |  | **Validation stage** | |  | **Meta-analysis** | |
|  |  | **β (SE)** | ***P*** |  | **β (SE)** | ***P*** |  | **β (SE)** | ***P*** |
| cg03418289 |  | 0.77 (0.27) | 3.93E-03 |  | 0.44 (0.42) | 2.91E-01 |  | 0.67 (0.22) | 2.72E-03 |
| **cg12593793** |  | **-0.85 (0.17)** | **5.13E-07** |  | **-1.02 (0.39)** | **9.46E-03** |  | **-0.88 (0.16)** | **1.71E-08** |
| cg24674445 |  | -0.83 (0.29) | 3.66E-03 |  | 0.44 (0.53) | 4.02E-01 |  | -0.54 (0.25) | 3.15E-02 |
| cg25570328 |  | -0.50 (0.18) | 4.97E-03 |  | -1.10 (0.32) | 7.49E-01 |  | -0.41 (0.16) | 9.13E-03 |
| cg06398474 |  | -0.83 (0.24) | 4.10E-04 |  | NA | NA |  | NA | NA |
| cg16984944 |  | -0.42 (0.22) | 5.84E-02 |  | -0.29 (0.37) | 4.37E-01 |  | -0.39 (0.19) | 4.33E-02 |
| cg01741041 |  | 0.60 (0.21) | 3.58E-03 |  | -0.58 (0.42) | 1.69E-01 |  | 0.37 (0.18) | 4.32E-02 |
| cg07914621 |  | -0.33 (0.27) | 2.13E-01 |  | -0.53 (0.58) | 3.58E-01 |  | -0.37 (0.24) | 1.29E-01 |
| cg01866330 |  | 0.49 (0.30) | 1.07E-01 |  | -0.57 (0.54) | 2.93E-01 |  | 0.24 (0.26) | 3.71E-01 |
| cg25392060 |  | 0.87 (0.25) | 4.78E-04 |  | 0.30 (0.50) | 5.45E-01 |  | 0.76 (0.22) | 6.74E-04 |
| **cg17061862** |  | **-0.77 (0.25)** | **1.94E-03** |  | **-1.04 (0.52)** | **4.32E-02** |  | **-0.82 (0.22)** | **2.42E-04** |
| cg00574958 |  | -0.48 (0.25) | 9.99E-02 |  | -0.95 (0.49) | 5.52E-02 |  | -0.60 (0.25) | 1.68E-02 |
| cg00862597 |  | 0.44 (0.21) | 3.98E-02 |  | 0.40 (0.42) | 3.36E-01 |  | 0.43 (0.19) | 2.33E-02 |
| cg02484673 |  | -0.37 (0.20) | 6.90E-02 |  | 0.21 (0.38) | 5.77E-01 |  | -0.24 (0.18) | 1.82E-01 |
| cg23415756 |  | 0.88 (0.30) | 2.93E-03 |  | -0.16 (0.45) | 7.15E-01 |  | -0.66 (0.25) | 7.33E-03 |
| **cg11024682** |  | **0.88 (0.23)** | **1.37E-04** |  | **1.51 (0.44)** | **6.25E-04** |  | **1.02 (0.20)** | **6.98E-07** |
| cg23985214 |  | 0.67 (0.26) | 8.78E-03 |  | 0.21 (0.43) | 6.21E-01 |  | 0.55 (0.22) | 1.22E-02 |
| cg23510258 |  | 0.67 (0.19) | 4.04E-04 |  | 0.53 (0.44) | 2.34E-01 |  | 0.65 (0.18) | 2.00E-04 |
| cg07769588 |  | 0.59 (0.25) | 1.67E-02 |  | -0.60 (0.54) | 2.60E-01 |  | 0.38 (0.22) | 8.73E-02 |
| **cg06500161** |  | **1.63 (0.29)** | **2.14E-08** |  | **1.63 (0.52)** | **1.71E-03** |  | **1.63 (0.25)** | **1.38E-10** |
| **Note:** Association analyses were performed separately in each sub-study using linear regression models, with inverse-normal transformed DNA methylation levels included as dependent variables, natural logarithm transformed BMI as the independent variable, with adjustment for age, gender, drinking status and all SVs. | | | | | | | | | |

| **Table S4. Methylation-gene expression correlations of the 4 BMI-related CpGs in SY-EXPR population (n=144).** | | | | | | | | | | | | |
| --- | --- | --- | --- | --- | --- | --- | --- | --- | --- | --- | --- | --- |
| **CpG information** | | | | | |  | **Transcript information** | |  | **CpG-expression association** | | |
| **Chr** | **Position** | **CpGs** | **Gene** | **Relation to Gene** | **Nearest CpG islands** |  | **Probe** | **Gene** |  | **β (SE)** | ***P*** | **Expression rate** |
| 1 | 156074135 | cg12593793 | *LMNA* | body |  |  | ILMN_1696749 | *LMNA* |  | -0.50 (1.76) | 8.3E-01 | 100.00% |
|  |  |  |  |  |  |  | ILMN_1737394 | *LMNA* |  | -1.70 (1.76) | 4.5E-01 | 100.00% |
| 11 | 9590431 | cg17061862 | *ZNF143* | <50kb | Chr11: 9594346-9596536 |  | ILMN_1674399 | *ZNF143* |  | 0.58 (1.84) | 7.7E-01 | 100.00% |
| 17 | 17730094 | cg11024682 | *SREBF1* | body | Chr17: 17726855-17727282 |  | ILMN_1663035 | *SREBF1* |  | -7.30 (3.76) | **3.0E-03** | 100.00% |
|  |  |  |  |  |  |  | ILMN_2328986 | *SREBF1* |  | -6.38 (3.76) | 1.0E-02 | 100.00% |
| 21 | 43656587 | cg06500161 | *ABCG1* | body | Chr21: 43654846-43655465 |  | ILMN_1658176 | *ABCG1* |  | -7.39 (3.31) | 2.2E-02 | 100.00% |
|  |  |  |  |  |  |  | ILMN_2329927 | *ABCG1* |  | -11.76 (3.19) | **1.9E-04** | 100.00% |
|  |  |  |  |  |  |  | ILMN_1794782 | *ABCG1* |  | -10.67 (3.23) | **7.8E-04** | 100.00% |
| **Note:** Methylation-expression correlations were calculated using linear regressions in which inverse-normal transformed expression values were regressed on original methylation values adjusted for age and gender. | | | | | | | | | | | | |

| **Table S5. Estimated OR and 95%CI for NSCLC risk per SD increase in DNA methylation levels of 4 BMI-related CpGs after adjustment for major leukocyte counts.** | | | | | | | | | |
| --- | --- | --- | --- | --- | --- | --- | --- | --- | --- |
| **CpGs** | **NSCLC-1** | |  | **NSCLC-2** | |  | **Meta-analysis** | | |
|  | **OR (95%CI)** | ***P*** |  | **OR (95%CI)** | ***P*** |  | **OR (95%CI)** | ***P*** | ***P*_heterogeneity_** |
| cg12593793 | 0.18 (0.11, 0.31) | 8.99E-10 |  | 0.22 (0.14, 0.34) | 7.67E-11 |  | 0.20 (0.14, 0.29) | 2.38E-19 | 6.39E-01 |
| cg17061862 | 1.10 (0.80, 1.49) | 5.70E-01 |  | 1.33 (0.98, 1.81) | 7.13E-02 |  | 1.21 (0.97, 1.50) | 1.02E-01 | 4.01E-01 |
| cg11024682 | 0.46 (0.31, 0.67) | 5.36E-05 |  | 0.28 (0.18. 0.43) | 4.16E-09 |  | 0.37 (0.28, 0.49) | 5.98E-12 | 9.20E-02 |
| cg06500161 | 0.74 (0.54, 1.01) | 6.03E-02 |  | 0.61 (0.46, 0.82) | 1.09E-03 |  | 0.67 (0.54, 0.83) | 2.47E-04 | 3.86E-01 |
| **Note:** Association analyses were performed separately in each lung cancer case-control study using logistic regression models, with lung cancer status as the dependent variable, inverse-normal transformed methylation levels as the independent variables, and age, gender, BMI, smoking status, drinking status, and major leukocyte counts [including neutrophils, lymphocytes, and intermediate cells (the sum of monocytes, eosinophils and basophils)] as covariates. Results were combined using fixed-effect meta-analysis. | | | | | | | | | |

| **Table S6. Estimated OR and 95%CI for NSCLC risk per SD increase in the methylation levels of 4 BMI-related CpGs after stratified by the smoking status.** | | | | | | | | | | | | |
| --- | --- | --- | --- | --- | --- | --- | --- | --- | --- | --- | --- | --- |
| **CpG** | **Smoking status** | **NSCLC-1** | | |  | **NSCLC-2** | | |  | **Meta-analysis** | | |
|  |  | **Cases/**  **Controls, n** | **OR (95%CI)** | ***P* value** |  | **Cases/**  **Controls, n** | **OR (95%CI)** | ***P* value** |  | **Cases/**  **Controls, n** | **OR (95%CI)** | ***P* value** |
| cg12593793 |  |  |  |  |  |  |  |  |  |  |  |  |
|  | never | 40/56 | 0.22 (0.11, 0.46) | 4.15E-05 |  | 54/70 | 0.21 (0.11, 0.40) | 2.51E-06 |  | 94/126 | 0.21 (0.13, 0.35) | 6.29E-10 |
|  | ever | 65/47 | 0.15 (0.07, 0.32) | 7.46E-07 |  | 71/53 | 0.29 (0.17, 0.49) | 6.32E-06 |  | 136/100 | 0.23 (0.15, 0.36) | 3.86E-11 |
| cg17061862 |  |  |  |  |  |  |  |  |  |  |  |  |
|  | never | 40/56 | 0.78 (0.50, 1.22) | 2.81E-01 |  | 54/70 | 1.04 (0.68, 1.61) | 8.40E-01 |  | 94/126 | 0.90 (0.66, 1.24) | 5.35E-01 |
|  | ever | 65/47 | 1.46 (0.95, 2.25) | 8.27E-02 |  | 71/53 | 1.40 (0.93, 2.12) | 1.07E-01 |  | 136/100 | 1.49 (1.11, 2.00) | 1.80E-02 |
| cg11024682 |  |  |  |  |  |  |  |  |  |  |  |  |
|  | never | 40/56 | 0.95 (0.35, 0.98) | 3.64E-02 |  | 54/70 | 0.36 (0.21, 0.62) | 1.93E-04 |  | 94/126 | 0.47 (0.32, 0.68) | 6.42E-05 |
|  | ever | 65/47 | 0.30 (0.17, 0.54) | 4.75E-05 |  | 71/53 | 0.35 (0.21, 0.58) | 2.53E-05 |  | 136/100 | 0.33 (0.22, 0.47) | 7.71E-09 |
| cg06500161 |  |  |  |  |  |  |  |  |  |  |  |  |
|  | never | 40/56 | 0.75 (0.46, 1.22) | 2.44E-01 |  | 54/70 | 0.61 (0.40, 0.94) | 2.70E-02 |  | 94/126 | 0.67 (0.48, 0.94) | 1.48E-02 |
|  | ever | 65/47 | 0.69 (0.40, 1.05) | 8.53E-02 |  | 71/53 | 0.60 (0.40, 0.91) | 1.46E-02 |  | 136/100 | 0.64 (0.48, 0.86) | 3.05E-03 |
| **Note:** Association analyses were performed separately in each stratum by using multiple logistic regression models, with lung cancer status as dependent variable, inverse-normal transformed methylation beta values as the independent variable, with adjustment for age, gender, BMI and drinking status. Results were combined using fixed-effect meta-analysis. | | | | | | | | | | | | |


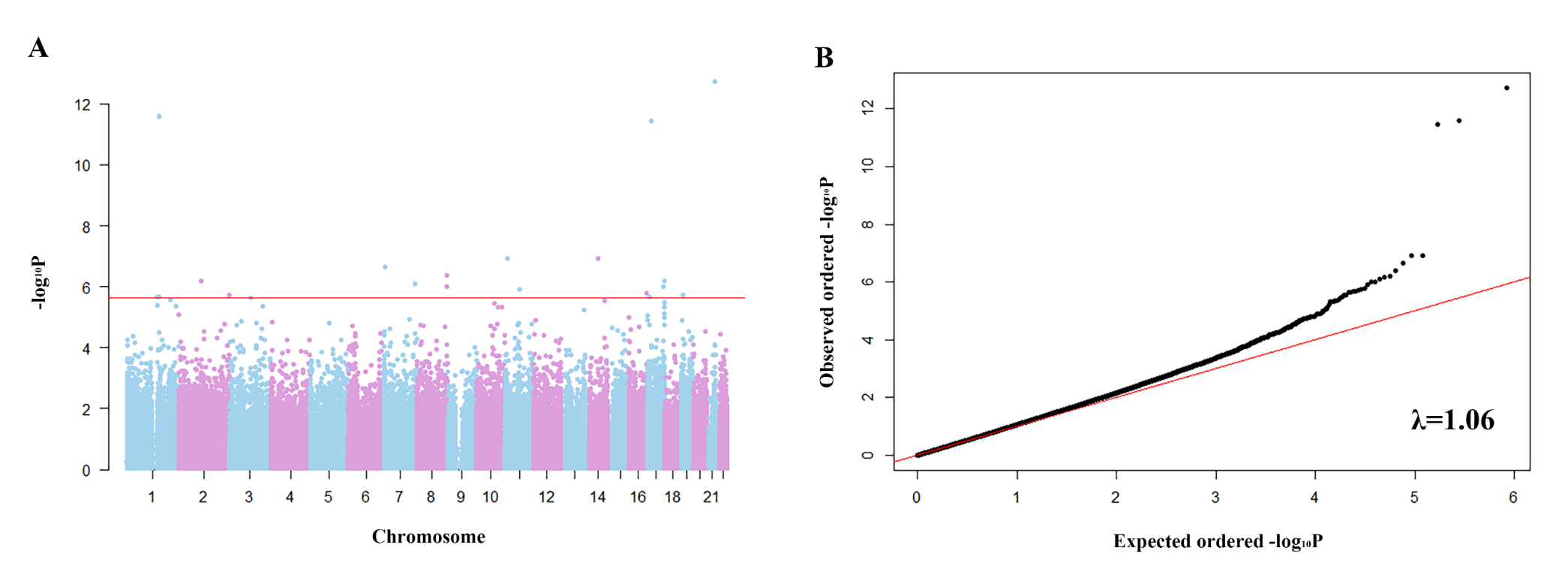


**Figure S1.** Epigenome-wide meta-analysis of body mass index (BMI) in the discovery stage.

(A) Manhattan plot, all points above the solid line are at FDR<0.05 (20 CpGs).

(B) Quantile-quantile plot of the EWAS results.

**Note:** A fixed-effect meta-analysis of EWAS weighted on the inverse variance was performed to establish the observational association between BMI and differential DNA methylation in the discovery stage.

**
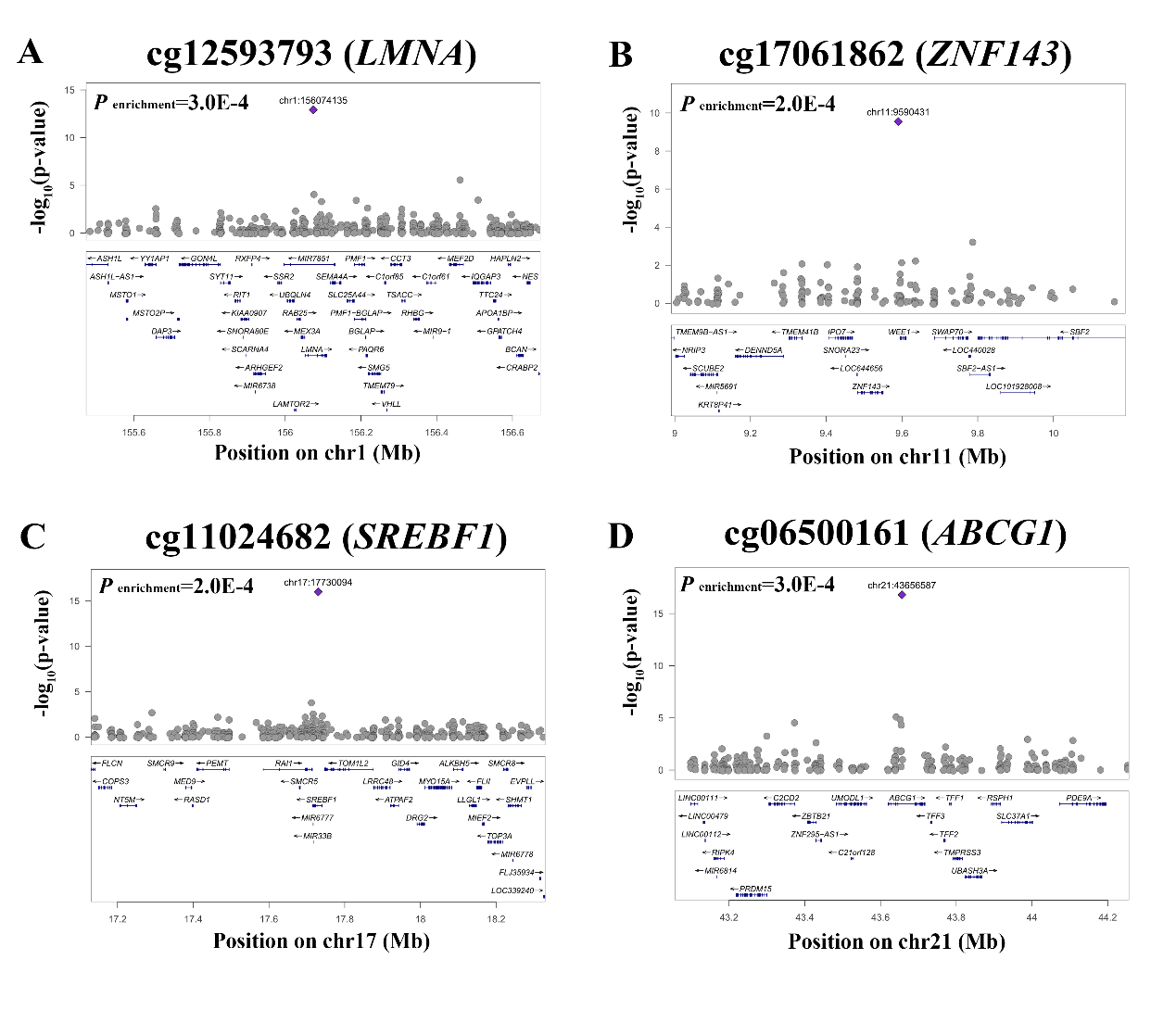
**

**Figure S2.** Associations of all methylation sites (±600 kbps) surrounding the 4 BMI-related CpGs with BMI.

**Note:** This plot was depicted based on the meta-analysis of both discovery stage and validation stage. The horizontal axis denotes the position of gene, and the vertical one denotes the value of -log_10_*P*. The diamond nodes represented the discovered and replicated 4 CpGs, and the dots represented the methylation sites (±600 kbps) surrounding the 4 CpGs.


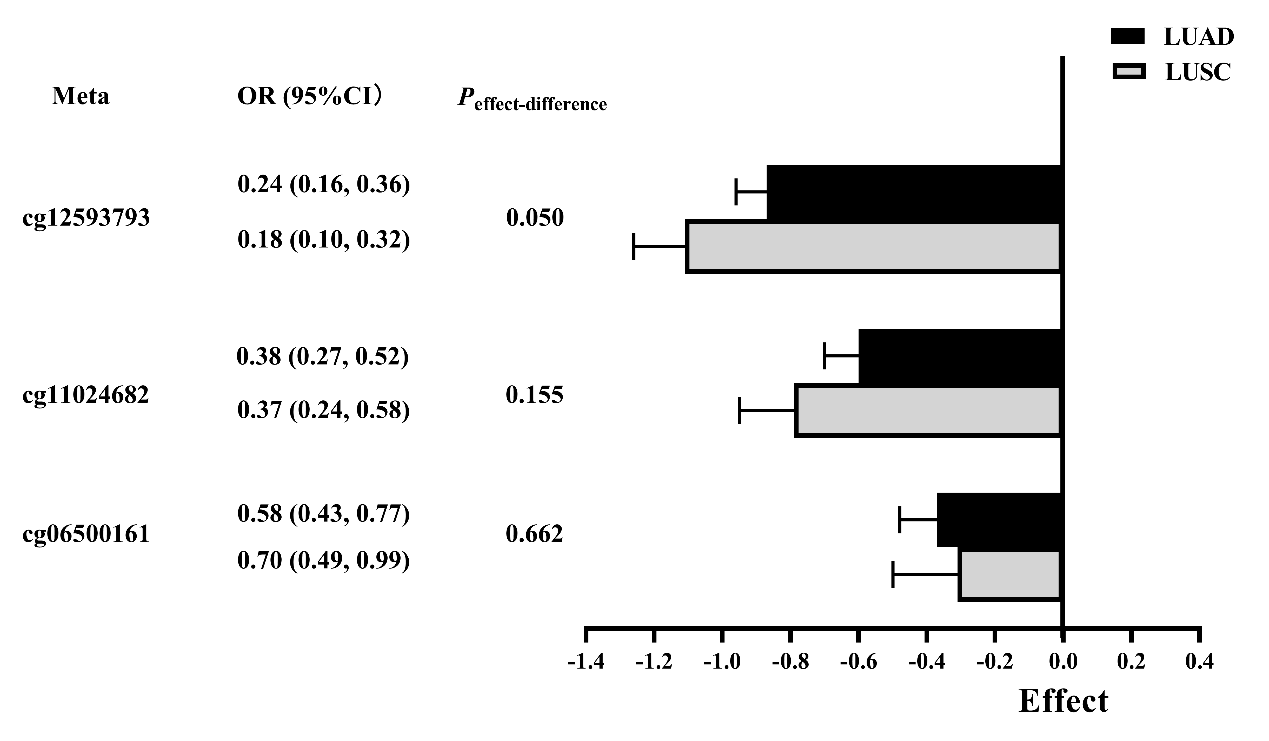


**Figure S3.** Comparison of effect sizes between lung adenocarcinoma (LUAD) and lung squamous carcinoma (LUSC) for the three CpGs (cg12593793, cg11024682 and cg06500161) related to both BMI and lung cancer risk in NSCLC-1 and NSCLC-2 subsets.

**Note:** Black bars stand for effect sizes of LUAD and gray bars correspond to effect sizes of LUSC.
